# What prognostic information does flow cytometry provide in canine B-cell lymphoma?

**DOI:** 10.18849/ve.v7i1.381

**Published:** 2022-03-24

**Authors:** Benjamin Haythornthwaite+

**Keywords:** B-CELL, FLOW CYTOMETRY, IMMUNOPHENOTYPING, LYMPHOMA, PROGNOSIS

## Abstract

In dogs with B-cell lymphoma, does the use of flow cytometry provide useful prognostic information?

Clinical bottom line

Category of research question

Prognosis

The number and type of study designs reviewed

Twelve papers were critically reviewed. All were cohort studies

Strength of evidence

Moderate

Outcomes reported

There are multiple potential prognostic indicators in canine B-cell lymphoma that flow cytometry can be used to evaluate, including lymphoma stage, survival time and time to progression. There is promising evidence for the use of percentage expression of CD25 and Ki67 cellular markers in providing prognostic information in canine B-cell lymphoma and these should be assessed further in clinical practice. Flow cytometry has also been shown to be useful in assessing bone marrow infiltration and providing prognostic information relating to this. There is also evidence for the prognostic value of measuring expression of class II MHC and CTLA-4 cellular markers, peripheral lymphocyte / monocyte ratio, nodal regulatory T-cell populations and the ratio between T and B lymphocytes in extranodal locations. Peripheral regulatory T-cell populations and cellular size were also assessed, however further investigations are required before confirming their prognostic value

Conclusion

Flow cytometric analysis offers useful measures of prognosis in canine B-cell lymphoma, although further validation is required before introducing their routine use. Percentage expression of CD25 and Ki67 cellular markers from lymph node aspirates of dogs with B-cell lymphoma appear to be promising prognostic indicators in clinical investigations, however this needs to be translated into clinical practice. While there is evidence for the prognostic value of bone marrow infiltration measured flow cytometrically, expression of class II MHC and CTLA-4, peripheral lymphocyte / monocyte ratio, nodal regulatory T-cell populations and the ratio between T and B lymphocytes in extranodal locations, these need to be further investigated before introducing into clinical practice. As new antibodies against cellular targets in dogs become available, it is likely that flow cytometry will become even more useful in providing prognostic information

## How to apply this evidence in practice

The application of evidence into practice should take into account multiple factors, not limited to: individual clinical expertise, patient’s circumstances and owners’ values, country, location or clinic where you work, the individual case in front of you, the availability of therapies and resources.

Knowledge Summaries are a resource to help reinforce or inform decision making. They do not override the responsibility or judgement of the practitioner to do what is best for the animal in their care.

## The evidence

Twelve studies in total were found relevant to the PICO, all of which were cohort studies (Baek et al., 2017; Joetzke et al., 2012; Marconato et al., 2013; Marconato et al., 2015; Mizutani et al., 2016; Pinheiro et al., 2014; Poggi et al., 2015, Poggi et al., 2017; Rao et al., 2011; Riondato et al., 2021; Tagawa et al., 2018; and Wolf-Ringwall et al., 2019). Eight of the studies looked to correlate flow cytometric analysis with survival data (Marconato et al., 2013; Marconato et al., 2015; Mizutani et al., 2016; Pinheiro et al., 2014; Poggi et al., 2017; Rao et al., 2011; Tagawa et al., 2018; and Wolf-Ringwall et al., 2019). One study looked to correlate stage of B-cell lymphoma with percentage of Tregs in peripheral blood measured by flow cytometry (Baek et al., 2017).  One study looked to correlate flow cytometric data with involvement of lymphoma in extranodal tissues (Joetzke et al., 2012). Two studies looked to correlate Ki67 expression with grade of lymphoma (Poggi et al., 2015; and Riondato et al., 2021).

All studies were observational in nature which is likely suitable for this PICO question, however more prospective studies may be useful in the future to produce more robust studies with fewer sources of potential bias or confounding factors. Two studies had more than 100 patients (Rao et al., 2011; and Riondato et al., 2021). Three studies had between 50 and 100 patients (Marconato et al., 2015; Poggi et al., 2015; and Wolf-Ringwall et al., 2019). The other seven studies had less than 50 patients (Baek et al., 2017; Joetzke et al., 2012; Marconato et al., 2013; Mizutani et al., 2016; Pinheiro et al., 2014; Poggi et al., 2017; and Tagawa et al., 2018).  Studies were generally well constructed and provided strong evidence towards the answering of the PICO question. This will be discussed in the appraisal.

## Summary of the evidence

**Baek et al. (2017):** 

Population:	Dogs with B-cell lymphoma.
Sample size:	12 dogs with B-cell lymphoma. Five healthy Beagles used as a control.
Intervention details:	Dogs with B-cell lymphoma were classified based on the World Health Organization (WHO) clinical staging system for lymphosarcoma in domestic animals (Owen, 1980). 10 dogs with lymphoma were classified as stage IV, and two were classified as stage V. Peripheral blood samples were taken from the study patients and peripheral blood mononuclear cells were assessed for the expression of cell markers of regulatory T-cells (Tregs) measured by flow cytometry. The extracellular markers CD4 and CD25 were used as well as the intracellular marker FoxP3. The percentage of Tregs was calculated by dividing the CD4+CD25+FoxP3+ T-cells by the total number of CD4+ T-cells. The total number of lymphocytes in the blood samples of each patientwas determined by a routine complete blood count and a differential count on a blood smear ― this was then used to calculate the number of Tregs with the percentage of Tregs found above.
Study design:	Cohort study.
Outcome studied:	Percentage of Tregs measured by flow cytometry in peripheral blood of the patients. Patients with B-cell lymphoma were compared to the average percentage of Tregs in the five healthy controls (5.2%). Patients with WHO clinical stage IV lymphoma were compared to patients with WHO clinical stage V lymphoma.
Main Findings (relevant to PICO question)	All 12 canine patients with B-cell lymphoma showed significantly increased percentages of Tregs (mean 19.6%) compared to the average percentage of Tregs in the five healthy controls (5.2%). The absolute numbers of Tregs in the peripheral blood of the dogs with B-cell lymphoma (mean 1342 × 103 cells / μl blood) was also significantly increased compared to the five healthy controls (51 × 103 cells / μl blood). Dogs in WHO clinical stage V showed increased relative (57.2% vs 16.2%) and absolute (5809 × 103 / μl vs 936 × 103 / μl) numbers of Tregs when compared to dogs in WHO clinical stage IV.
Limitations:	Small sample size. Breeds of dogs with lymphoma not described. It is not mentioned how the diagnosis of lymphoma was confirmed in sample patients. WHO clinical stages sampled only included stages IV and V and it was not explicitly explained how staging was performed and differed between them. Stage migration depending on staging technique may impact how these results are interpreted. Only two WHO clinical stage V patients were sampled, compared to ten WHO clinical stage IV patients. Not directly associated with prognosis but indicates a potential prognostic target.

**Joetzke et al. (2012):** 

Population:	Dogs with cytologically confirmed multicentric B- or T-cell lymphoma. A diagnosis of B-cell or T-cell lymphoma was required to be supported by flow cytometric evaluation of fine needle aspirates of lymph nodes with antibodies against CD21 or CD3 respectively.
Sample size:	44 dogs from 21 different breeds with multicentric B-cell (n = 35) or T-cell (n = 9) lymphoma. Five healthy control Beagles.
Intervention details:	Clinical staging of the dogs with lymphoma was performed according to the World Health Organization clinical staging system (Owen, 1980). Samples were taken from blood, bone marrow, liver and spleen. Cell counts in all samples were measured with an automated blood cell counter. Nucleated cells were then assessed for the cell markers CD45, CD3 and CD21 by flow cytometry. The percentages of CD3+CD21- (T-cells) and CD21+CD3- (B-cells) were used to calculate T:B values. Mean values of the log (T:B) ±2 standard deviations in the healthy control dogs were used to define upper and lower threshold values for each sample type. Samples from the patients with T-cell lymphoma would be considered positive for lymphoma involvement if the log (T:B) from the sample was greater than the upper threshold value. Samples from the patients with B-cell lymphoma would be considered positive for lymphoma involvement if the log (T:B) from the sample was less than the lower threshold value.
Study design:	Cohort study.
Outcome studied:	Involvement of lymphoma in extranodal tissues as defined in the intervention details.
Main Findings (relevant to PICO question)	In dogs with B-cell lymphoma, mean log (T:B) values of lymph node (-1.11 ± 0.47 vs 0.45 ± 0.20), blood (0.12 ± 0.64 vs 0.85 ± 0.15), bone marrow (-0.22 ± 0.59 vs 0.40 ± 0.49), liver (-0.24 ± 0.61 vs 1.08 ± 0.27) and spleen (-0.93 ± 0.44 vs 0.33 ± 0.18) samples were significantly lower than those in samples of the same tissue type from the healthy control dogs (P<0.001 in lymph node, blood, liver and spleen samples; P = 0.017 in bone marrow). On the basis of the defined thresholds results were positive for 25/35 (71.4%) blood samples, 11/34 (32.4%) bone marrow samples, 26/29 (89.7%) of liver samples and 34/34 (100.0%) of spleen samples from dogs with B-cell lymphoma. In 30/35 (86.0%) of the dogs with B-cell lymphoma, a population of presumed neoplastic B-cells was identified at more than one sample site by flow cytometry. In the other five, apparently normal B-cells were depleted in all sample sites or a confluence of presumed neoplastic and non-neoplastic B-cells to one indistinguishable population. In 24/35 (69.0%) of dogs with B-cell lymphoma, relative CD21 expression density was higher in presumed neoplastic than in non-neoplastic lymphocytes in more than one sample. Results of cytologic and flow cytometric examination were in agreement for 133/161 (83.0%) of sample collection sites.
Limitations:	Dogs from a large range of breeds were used in different numbers. Only five normal dogs were used to calculate the upper and lower threshold values for each sample type which may not be fully representative. A small number of antibodies were used to classify lymphocytes. Blood contamination may have led to false classifications in cytologic and flow cytometric examinations. Lymphocyte populations detected in samples with light scatter and antibody binding characteristics similar to lymphoma cell populations in the lymph nodes of the same patient can only be assumed to be neoplastic – it cannot be ruled out that these are physiologic lymphocyte subpopulations. Not directly associated with prognosis but indicates a potential prognostic target.

**Marconato et al. (2013):** 

Population:	Dogs with multicentric large B-cell lymphoma diagnosed by cytology and flow cytometry or histopathology. Dogs were enrolled from 2007 to 2012.
Sample size:	46 dogs total from 21 different breeds.
Intervention details:	All dogs were staged using the World Health Organization (WHO) system (Owen, 1980). Flow cytometric immunophenotyping was carried out on lymph node aspirates, bone marrow aspirates and peripheral blood of each dog at the time of initial staging. A panel of antibodies was used to assess the presence of the following cell markers using a multi-colour approach: CD45, CD3, CD5, CD4, CD8, CD21, CD79a and CD34. The extent of peripheral blood and bone marrow infiltration by large B-cells was evaluated by flow cytometry. This was reported as a percentage of large CD21+ positive cells out of total CD45 positive cells. Additionally, in dogs recruited after June 2011 (n = 13) histopathology was performed on the same lymph node that was aspirated for cytology and flow cytometry. The lymph node was examined by a veterinary pathologist and antibodies against CD3, CD5, CD79a and CD20 were used for immunohistochemistry on paraffin embedded sections. Treatment was then initiated using the following chemotherapeutic protocol: Day 1: L-asparaginase Day 8: Vincristine Days 15–18: Cyclophosphamide Day 22: Doxorubicin The cycle was repeated once after a 1-week interval. A D-actinomycin, cytarabin and melphalan protocol was offered in case of relapse. Response to treatment was evaluated by assessment of peripheral lymph nodes 24 hours starting after the first administration of L-asparaginase and at the end of each session of chemotherapy after this. Response was classified as complete remission, partial remission, stable disease or progressive disease – responses were required to last for ≥ 28 days. Dogs were re-evaluated at the end of chemotherapy. Any investigations that previously had abnormal results in the pre-treatment stages were repeated. Flow cytometry was repeated on bone marrow and peripheral blood samples in all cases at the end of chemotherapy.
Study design:	Cohort study.
Outcome studied:	Time to progression was calculated from the start of treatment to progressive disease. Lymphoma specific survival was measured as the interval between the start of treatment and death from lymphoma. The authors looked for an association between peripheral blood or bone marrow infiltration assessed by flow cytometry and time to progression or lymphoma specific survival.
Main Findings (relevant to PICO question)	At the end of the study 6/46 (13.0%) dogs were alive and in complete remission, while 40/46 (87.0%) had died. Of these, 38/46 (82.6%) died because of progressive disease and 2/46 (4.3%) died from causes unrelated to lymphoma. Bone marrow infiltration significantly influenced time to progression (P = 0.001) on the basis of univariable Cox’s proportional hazard regression. Bone marrow infiltration significantly influenced lymphoma specific survival (P<0.001) on the basis of univariable Cox’s proportional hazard regression. A cut-off of 0% bone marrow infiltration was proposed to identify dogs with an unfavourable prognosis – hazard ratios showed that dogs with ≥3.0% bone marrow infiltration had a 3.3 (95.0% CI 1.4–7.6) times higher probability of progression, and a 3.6 (95.0% CI 1.6–8.0) times higher probability of death, compared to dogs with <3.0% bone marrow infiltration. Although significant values were also found when using a cut-off of 1.0% bone marrow infiltration, this is very close to the intrinsic limit of detection of flow cytometry as large B-cells have also been found in healthy dogs at a percentage close to but less than 1.0%. In the multivariate analysis, substage was the only factor associated with time to progression (P = 0.002), while both substage (P < 0.001) and anaemia (P = 0.008) were associated with lymphoma specific survival. Bone marrow infiltration was not found to be significant in the multivariable analysis, showing it was not an independent prognostic factor.
Limitations:	Histopathology was only performed on 13/46 (28.3%) of the study population. Some dogs received corticosteroids before initiation of the study protocol. Bone marrow aspiration was routinely unilateral therefore sensitivity for bone marrow involvement may have been decreased. Focal bone marrow infiltration may have been missed as trephine bone marrow biopsy was not performed. The decision whether to perform rescue therapy if required was left to the owners which may have biased lymphoma specific survival in some cases. Large confidence intervals were found for the hazard ratios. Bone marrow infiltration lost significance in the multivariable analysis, showing it is not an independent prognostic factor. Although explored in this study, further variables need to be better characterised.

**Marconato et al. (2015):** 

Population:	Dogs with diffuse large B-cell lymphoma diagnosed histologically and immunohistochemically.
Sample size:	51 dogs from 22 different breeds.
Intervention details:	All dogs were staged using the World Health Organization (WHO) system (Owen, 1980). Flow cytometric analysis of blood was used to assess lymphocyte and monocyte percentages by taking into account morphological scattergrams and the expression of CD45 (pan-leucocyte marker) and CD21 (B-cell marker). Absolute lymphocyte count (ALC) and absolute monocyte count (AMC) were calculated based on the white blood cell count from a complete blood count performed on the day of diagnosis and the percentages of lymphocytes and monocytes obtained by flow cytometric analysis. Lymphocyte/monocyte ratio (LMR) was calculated as the ratio of absolute counts between peripheral lymphocytes and monocytes. Treatment was then initiated using a cyclophosphamide, doxorubicin, vincristine and prednisolone (CHOP) based protocol with incorporation of Apavac immunotherapy. Response to treatment was assessed at each treatment session. Response was classified as complete remission, partial remission, stable disease or progressive disease - responses were required to last for ≥28 days. Dogs were re-evaluated at the end of chemotherapy. Any investigations that previously had abnormal results in the pre-treatment stages were repeated. Peripheral blood and bone marrow was sample again for flow cytometric analysis in all cases. All dogs were followed up monthly for the first year following this, then every 2 months. Rescue treatment was offered in case of relapse.
Study design:	Cohort study.
Outcome studied:	Time to progression was calculated from the start of treatment to progressive disease. Lymphoma specific survival was measured as the interval between the start of treatment and death from lymphoma. The authors looked for an association between LMR and time to progression or lymphoma specific survival.
Main Findings (relevant to PICO question)	During the study period, 38/51 (74.5%) dogs progressed and 13/51 (25.5%) dogs never relapsed. LMR was found to significantly influence time to progression for the 10th (P = 0.038), 20th (P = 0.003), 25th (P = 0.009) and 30th (P = 0.001) percentiles in the univariable analysis. The probability of progression was 3.691 (1.706–7.985) times higher in dogs with LMR ≤2 compared with dogs with LMR >1.2. LMR was found to significantly influence lymphoma specific survival for the 10th (P = 0.022), 20th (P = 0.004), 25th (P = 0.005) and 30th (P = 0.002) percentiles in the univariable analysis. The probability of death due to lymphoma was 4.131 (1.719–9.931) times higher in dogs with LMR ≤ 1.2 compared with dogs with LMR >1.2.
Limitations:	No healthy control population to compare normal LMR. Lymphocytes and monocytes were categorised based only on expression of CD45 and morphological properties, not making use of further markers to help better characterise the cells. The decision whether to perform rescue therapy if required was left to the owners which may have biased lymphoma specific survival in some cases. Relatively short median follow-up time.

**Mizutani et al. (2016):** 

Population:	Dogs with lymphoid malignancies diagnosed by cytology and / or histopathology.
Sample size:	26 dogs with lymphoid malignancies including high-grade B-cell lymphoma (n = 17), T zone lymphoma (n = 5), follicular lymphoma (n = 2), cutaneous lymphoma (n = 2) and acute lymphoblastic leukaemia (n = 3). Seven healthy dogs and six dogs with reactive lymphadenopathy were also sampled for comparison.
Intervention details:	Samples were taken from peripheral lymph nodes in the 24 nodal lymphoma cases, and from cutaneous masses in the 2 cutaneous lymphoma cases. Peripheral blood was separated by Ficoll-Paque gradient centrifugation from the 3 acute lymphoblastic leukaemia cases. The samples were stained for the CD25 marker, analysed by flow cytometry, and the percentages of CD25 positive cells were calculated from an isotype matched control for each sample. Further to this, 15 dogs with high-grade B-cell lymphoma were divided into two groups: >60.0% CD25+ cells (CD25 high) (n = 7) and <60.0% CD25+ cells (CD25 low) (n = 8). Dogs in both groups received a CHOP based protocol and were monitored following treatment for progression of disease or death. Progression free survival was calculated from the date of initial treatment to disease progression or death from any cause. Overall survival was calculated from the date of initial treatment to death from any cause.
Study design:	Cohort study.
Outcome studied:	Percentage of CD25+ cells in the samples. The authors also looked to correlate high or low percentage of CD25+ cells in a sample with progression of disease and survival.
Main Findings (relevant to PICO question)	Percentage of CD25+ cells were significantly higher in cases with high-grade B-cell lymphoma than in healthy dogs. CD25 positivity was variable in the patients with high-grade B-cell lymphoma (range 0.4%–97.1%). Response to treatment was not significantly different between CD25 high and CD25 low dogs. The median value of progression free survival in CD25 high dogs which was significantly shorter (P < 0.05) than in CD25 low dogs (28 days vs 140 days). Kaplan-Meier curves appeared to show a decreased overall survival of dogs in the CD25 high group compared to the CD25 low group (median: 115 days vs 244 days), but this did not quite reach statistical significance (P = 0.074).
Limitations:	Small sample size, especially for comparing between the CD25 high and CD25 low groups. Progression free survival and overall survival calculated from death of any cause, not limited to lymphoma related deaths. It is not explained how frequently dogs were assessed for progression of disease when comparing prognosis.

**Pinheiro et al. (2014):** 

Population:	Dogs with B-cell lymphoma diagnosed by cytology and / or histopathology.
Sample size:	22 dogs with B-cell lymphoma. 14 control dogs with T-cell lymphoma. 14 control dogs with reactive lymph node hyperplasia. Six control dogs with metastatic mast cell tumours. 25 healthy control dogs.
Intervention details:	All dogs with lymphoma were staged according to the WHO staging system (Owen, 1980). Samples were taken from affected lymph nodes of study dogs. Samples were assessed by flow cytometry for the cellular markers CD5, CD4, CD8, CD79b, CD21, CD34, class II MHC, FOXP3 and Helios. Cells were classified on the basis of the flow cytometric data. Patients were then treated using various chemotherapeutic protocols. In the B-cell lymphoma group: 14 dogs underwent a CHOP protocol. Three dogs underwent a COP protocol. One dog underwent a course of prednisolone. Three dogs were classified as ‘other’ (included cytarabine, L-asparaginase and lomustine. One dog did not receive chemotherapy. Time to remission, progression free survival and overall survival were calculated for each patient.
Study design:	Cohort study.
Outcome studied:	The B-cell lymphoma group was compared to the other groups which were regarded as controls. The authors looked to correlate FOXP3 and Helios expression (thereby measuring Helios-FOXP3+ Regulatory T-cells) and class II MHC expression with time to remission, progression free survival and overall survival.
Main Findings (relevant to PICO question)	Patients with a total FOXP3 expression less than or equal to the median value in the study (1.2%) survived longer than those with expression above the median value (322 days vs 169 days, P = 0.018). Patients with lower FOXP3 expression in the CD5+CD4+ T-cell population had a longer progression free survival than those with higher FOXP3 expression (211 days vs 61 days, P = 0.012). Patients with Helios expression less than or equal to the median value (3.0%) had a longer progression free survival than those with expression above the median value (175 days vs 62 days, P = 0.014). Class II MHC expression was expressed as a ratio of MHC II+:MHC II- cells within the CD5- subset. Time to remission was longer in cases with expression ratios less than or equal to the median value (4.40) compared to cases with expression ratios greater than the median value (22 days vs 10 days). In the multivariable analysis, FOXP3 expression was found to be associated with progression free survival and overall survival. Class II MHC expression was found to be associated with time to remission in the univariable analysis.
Limitations:	Various chemotherapeutic strategies used for dogs in the study. Some dogs included in the study had been pretreated with corticosteroids or another cytotoxic chemotherapeutic drug prior to the study, however this was taken into account in the study and was not thought to impact the results. Time to remission, progression free survival and overall survival were defined by owners and veterinarians observations of clinical signs and lymph node size. Some dogs received rescue therapy while others did not.

**Poggi et al. (2015):** 

Population:	Dogs with lymphoma diagnosed by clinical presentation, cytological examination and flow cytometric analysis.
Sample size:	90 dogs with lymphoma including 80 high-grade (62 B-cell and 18 T-cell) and 10 low-grade (3 B-cell and 7 T-cell).
Intervention details:	Fine needle aspirates were taken from lymph nodes of the patients and cells were assessed by flow cytometry for the presence of the following cell markers: CD3, CD5, CD4, CD8, CD21, CD79a and CD34 in order to classify the type of lymphoma. Cells were also assessed using for the presence of Ki67 using flow cytometry and proliferative activity was expressed as percentage of Ki67+ cells. Fine needle aspirates of enlarged lymph nodes were also used to produce smears for cytological examination. All cases were classified and allocated to the low-grade or high-grade group according to the updated Kiel scheme (Fournel-Fleury et al., 1997; and Ponce et al., 2010).
Study design:	Cohort study.
Outcome studied:	The authors looked to evaluate the accuracy of percentage of Ki67+ cells in discriminating high-grade and low-grade forms of lymphoma.
Main Findings (relevant to PICO question)	A significantly higher percentage of Ki67+ cells were found in the high-grade group (median 34.4%; range 7.8–85.0%) compared to the low-grade group (median 5.3%; range 1.3–11.4%) (P < 0.0001). ROC curve analysis identified a high accuracy of percentage of Ki67+ cells in discriminating between high-grade and low-grade lymphomas (area under the curve [AUC] = 99.4%). A cut-off value of 12.2% was indicated to detect high-grade lymphomas with a sensitivity of 96.3% and a specificity of 100.0%.
Limitations:	Much smaller sample size of dogs with low-grade lymphoma compared to dogs with high-grade lymphoma – for the purposes of this review article, there were only three low-grade B-cell lymphoma cases included. Not directly associated with prognosis but indicates a potential prognostic target. No survival data for the dogs so not really associated with prognosis directly.

**Poggi et al. (2017):** 

Population:	Dogs with high-grade B-cell lymphoma diagnosed by clinical presentation, cytological examination of lymph nodes and flow cytometric analysis.
Sample size:	40 dogs from 21 different breeds.
Intervention details:	Fine needle aspirates were taken from lymph nodes of the patients and cells were immunophenotyped by flow cytometry. Cells were assessed for the presence of the following cell markers: CD45, CD3, CD5, CD4, CD8, CD21, CD79b and CD34. Cells were also assessed using for the presence of Ki67 using flow cytometry and proliferative activity and was expressed as percentage of Ki67+ cells (Ki67%). Patients were divided into Ki67% groups: low if Ki67 ≤0%, intermediate if Ki67 between 20.1 and 40.0%, high if Ki67 > 40.0%. Fine needle aspirates of enlarged lymph nodes were also used to produce smears for cytological examination. All cases were classified and allocated to a specific grade of malignancy and cytological subtype according to the updated Kiel scheme (Fournel-Fleury et al., 1997; and Ponce et al., 2010). A UW-25 (CHOP based) chemotherapy protocol was initiated in all patients. Information was collected following this including achievement of remission, occurrence of relapse, survival at the end of the first chemotherapy protocol, lymphoma specific survival (time in days between the start of treatment and death from lymphoma), relapse free interval (time in days from when a dog achieved complete remission until relapse), date and cause of death. Responses were classified as complete remission, partial remission, stable disease or progressive disease. Relapses were treated with a second UW-25 protocol or L-asparaginase and lomustine.
Study design:	Cohort study.
Outcome studied:	The authors looked to assess if Ki67% correlated with clinical outcome judged by achievement of remission, occurrence of relapse, survival at the end of the first chemotherapy protocol, lymphoma specific survival and relapse free interval.
Main Findings (relevant to PICO question)	Ki67% showed near significant association with survival (P = 0.063) and achievement of complete remission (P = 0.075) at the end of the chemotherapy protocol. Percentages of both survival and complete remission were higher for dogs with intermediate Ki67% (85.7% and 81.0% respectively) compared to dogs with low Ki67% (50.0% and 33.0%) and high Ki67% (50.0% and 60.0%) treated with UW-25 protocol. Ki67% significantly influenced lymphoma specific survival (P=0.001) on univariable analysis and was confirmed to be an independent prognostic factor (P = 0.001) in the multivariable analysis. Dogs with intermediate Ki67% has significantly longer lymphoma specific survival (median = 866 days) than dogs with low (median = 42 days; P<0.001) and high (median = 173 days; P = 0.038) Ki67%. Intermediate Ki67% was a significant predictor for 1 and 2 year survival (P = 0.001 and P = 0.004 vs low and high Ki67%, respectively, at both time points). Dogs with intermediate Ki67% showed longer relapse free interval (median = 428 days) than dogs with low Ki67% (median = 159 days; P = 0.014) and high Ki67% (median = 100 days; P = 0.126), although the difference with the high Ki67% group did not reach statistical significance.
Limitations:	Limited number of cases. Multiple different classifications of B-cell lymphoma were included in this study. Rescue chemotherapy protocol varied depending on when the relapse occurred and owner compliance. Many breeds of dogs represented in the study population.

**Rao et al. (2011):** 

Population:	Dogs with B-cell lymphoma diagnosed cytologically or histologically.
Sample size:	160 dogs.
Intervention details:	Fine needle aspirates of peripheral lymph nodes were taken from each patient and analysed by flow cytometry. Patients were retrospectively assigned as class II MHC low or class II MHC high based on median fluorescence intensity. Cells were classified as either medium or large lymphocytes based on median forward scatter of CD21 gated cells (large cells had a median forward scatter >720U, the remaining cases were categorised as medium cells). Cases in which >5.0% of B-cells stained for CD34 above background isotype control were considered CD34+. Treatment was initiated in all patients. Patients received either prednisolone only, single agent doxorubicin (+- prednisolone) or a CHOP based protocol. Two outcomes were recorded: survival time (time interval from starting treatment to death) and first remission time (time from starting treatment to date of first confirmed recurrence of cancer).
Study design:	Cohort study.
Outcome studied:	The authors looked to correlate expression of class II MHC, CD34 and cell size with survival times and first remission times.
Main Findings (relevant to PICO question)	Level of CD34, CD5 and CD21 expression did not predict survival or remission. When taking into account other factors in the multivariable model, median survival time for dogs with low class II MHC expression was 120 days, compared with 314 days for dogs with high class II MHC expression. Patients with low class II MHC expression were found to be 2.9 times more likely to die (95.0% CI = 1.4–5.9) in any time period compared to those with high expression. Patients with large tumour cells were 2.8 times more likely to die (95.0% CI = 1.0–7.5) in any time period compared to those with small tumour cells. A statistical model was produced by the authors which can be used to predict outcome of dogs with B-cell lymphoma based on significant prognostic factors (age, class II MHC, cell size and treatment).
Limitations:	The initial round of recruitment of patients did not have enough CD34+ cases, therefore a second round of recruitment was carried out. Variation in chemotherapy protocols used for each patient. Insufficient numbers of cases for breeds other than Golden Retrievers and Labrador retrievers were recruited to be able to analyse separately. 28 patients had a histological diagnosis, but these were not subclassified. Wide confidence intervals as low numbers some of the groupings.

**Riondato et al. (2021):** 

Population:	Dogs with a diagnosis of nodal lymphoma with flow cytometric analysis of an enlarged peripheral lymph node.
Sample size:	128 dogs with lymphoma. 90 dogs had B-cell lymphoma. 38 dogs had T-cell lymphoma.
Intervention details:	Cases were categorised as high- or low-grade B- or T-cell lymphoma based on review of the cytological preparations. Proliferation rate (Ki67%) was measured as the percentage of Ki67+ cells out of total nucleated cells. Apoptotic rate (AnnV%) was measured as the percentage of cells staining positive for Annexin V (used to detect apoptosis) but negative for propidium iodide. Proliferation/apoptosis ratio (PAR) was calculated as Ki67%/AnnV%. Turnover index (TI) was calculated as Ki67% + AnnV%. Statistical analysis was then performed for each continuous variable within each lymphoma subgroup.
Study design:	Cohort study.
Outcome studied:	The authors looked to correlate Ki67%, AnnV%, TI and PAR to see if there was any difference between high- and low-grade lymphoma cases.
Main Findings (relevant to PICO question)	Ki67% was significantly lower in low-grade B-cell lymphomas than in high-grade B-cell lymphomas (P < 0.001). AnnV% did not significantly vary between high- and low-grade B-cell lymphomas. PAR was significantly lower in low-grade B-cell lymphomas than in high-grade B-cell lymphomas (P < 0.001). TI was significantly higher in high-grade B-cell lymphomas than in low-grade B-cell lymphomas (P < 0.001).
Limitations:	This study looked at how Ki67%, AnnV%, PAR and TI related to grade of B-cell lymphoma but not on the prognostic significance of this. Classification was done by a single observer, increasing the chance of misclassification. Breed and sex not known for all dogs, however compared to the sample size this likely had little impact. No samples were analysed from lymph nodes without lymphoma, limiting interpretation.

**Tagawa et al. (2018):** 

Population:	Dogs with high-grade multicentric B-cell lymphoma confirmed cytologically or histologically.
Sample size:	18 dogs with lymphoma. Nine healthy control dogs.
Intervention details:	All dogs with lymphoma were staged according to the WHO staging system (Owen, 1980). Samples were taken from peripheral blood and fine needle aspirates were taken from palpable lymph nodes. Cells were analysed by flow cytometry and were assessed for the following cell markers: CD4, CD8, programmed death-1 (PD-1) and cytotoxic T-lymphocyte-associated antigen-4 (CTLA-4). RT-PCR amplification was also performed to measure the levels of PD-L1, PD-L2, CD80 and CD86 mRNA. Treatment was then started: 10 dogs were treated with a CHOP based protocol. Five dogs were treated with prednisolone alone. Three dogs were treated with L-asparaginase. Survival time was then assessed for each dog, measured as the interval between the sampling day and death due to lymphoma.
Study design:	Cohort study.
Outcome studied:	Whether expression of PD-1 and CTLA-4 on peripheral CD4+ and CD8+ lymphocytes was different between dogs with high-grade B-cell lymphoma and healthy control dogs. Whether there is a correlation between expression of PD-1 and CTLA-4 on peripheral CD4+ and CD8+ lymphocytes and survival time in dogs with high-grade B-cell lymphoma.
Main Findings (relevant to PICO question)	The proportions of PD-1+ cells in CD4+ lymphocyte populations obtained from peripheral blood mononuclear cells and lymph node cells were significantly higher in the lymphoma group than in the control group (P<0.001). The proportion of CTLA-4+ cells in CD4+ lymphocyte populations obtained from peripheral blood mononuclear cells was significantly higher in the lymphoma group than in the control group (P = 0.018). The proportion of PD-1+ and CTLA-4+ cells in CD8+ lymphocyte populations obtained from peripheral blood mononuclear cells were not significantly higher in the lymphoma group than in the control group. Optimal cut-off values for expression of PD-1 and CTLA-4 on CD4+ and CD8+ lymphocytes were established. Dogs with levels below the cutoff value for CTLA-4 expression had a significantly longer survival time than dogs with values above the cutoff (multiple P values depending on sample and CD4+/CD8+). However, CD8+ CTLA-4+ lymphocytes from lymph node cells did not meet a minimum AUC of 0.7. Dogs with levels below the cutoff value for CD4+ PD-1+ lymphocytes from lymph node cells had a significantly longer survival time than dogs with values above the cutoff (P = 0.023). However, this did not meet a minimum AUC of 0.7. Except for this, the expression of PD-1 on lymphocytes did not correlate with survival time.
Limitations:	A small sample size was used for this study. Two dogs were treated with prednisolone before sampling was carried out. There was variation in the chemotherapy protocol used to treat the dogs in the study. The authors highlighted a lack of protein quantification of the immune checkpoint ligands. CD4 expression is not exclusive to T-cells.

**Wolf-Ringwall et al. (2019):** 

Population:	Dogs diagnosed with multicentric B-cell lymphoma diagnosed by cytology, histology and confirmed by flow cytometry.
Sample size:	64 dogs.
Intervention details:	All dogs were staged using the World Health Organization (WHO) system (Owen, 1980) and the attending clinician was required to assign an approximate stage and substage. Multiple fine needle aspirates from a representative lymph node and analysed by flow cytometry. Cells were assessed for size and for the expression of the following cell markers: class II MHC, CD21, CD22, CD5, CD45 and CD25 on the neoplastic B-cells, and percent CD4 infiltration. Cases were assigned to cell size category large if CD21+ lymphocytes had a median forward scatter >720U. The remaining cases were assigned as medium. Level of CD25 expression was measured as the percentage of B-cells expressing CD25. All patients had lymph node biopsies and blood collection prior to treatment and at disease progression which were histologically and immunohistologically analysed. All dogs were treated with a 19 week CHOP protocol. And response to therapy was determined using the Veterinary Cooperative Oncology Group v1.0 response evaluation criteria for lymphoma (Vail et al., 2010).
Study design:	Cohort study.
Outcome studied:	Objective response rate was defined as the sum of patients with a complete response or a partial response. Progression free survival time was defined as the time from initiation of the chemotherapeutic protocol to disease progression or death from any cause. Overall survival time was defined as the time from initiation of the chemotherapeutic protocol to death from any cause.
Main Findings (relevant to PICO question)	Patients with diffuse large B-cell lymphoma with a high percent of CD25+ B-cells had a significantly decreased progression free survival (P = 0.04) and overall survival time (P = 0.03) compared to dogs with a lower percent of CD25+ B-cells. Percentage of CD25 expression was found to be a prognostic factor for overall survival for dogs with diffuse large B-cell lymphoma on univariable analysis. Cell size was not correlated with outcome in diffuse large B-cell lymphoma. Class II MHC expression was not correlated with outcome in diffuse large B-cell lymphoma.
Limitations:	Information is not given as to how sampled lymph nodes were chosen as representative. Full staging was not performed in all dogs ― 23/64 dogs (35.9%) had thoracic radiographs, 22/64 (34.4%) had an abdominal ultrasound, none had their bone marrow sampled. Overall survival time and progression free survival were used which assess death from any cause, not just death resulting from lymphoma. Dogs lost to follow up were considered to have died of their disease if they were known to be out of remission at their last visit. Only two patients fell into the large cell size category, which may explain the absence of correlation with survival. Two dogs received a modified CHOP protocol with vinblastine instead of vincristine due to adverse effects. There was an inadequate sample size of the less common subtypes of B-cell lymphoma in order to investigate them fully.

## Appraisal, application and reflection

Flow cytometry is now widely used in order to help classify lymphoma (Regetti and Bienzle, 2011) which can provide information relating to recommended treatment protocols and to help inform on prognosis from knowledge about different classes of lymphoma. With increasing availability of flow cytometers and antibodies against species specific cell markers in the veterinary sector, we are becoming aware of more markers whose expression becomes deranged in canine lymphoma. More studies are targeting cell markers or other cellular characteristics in lymphoma which may be useful as prognostic indicators or therapeutic targets. The twelve cohort studies evaluated in this Knowledge Summary provide evidence for the use of a range of cellular markers and characteristics in providing useful prognostic information in canine lymphoma.

From the studies evaluated, eight correlated specific cellular markers measured by flow cytometry with prognosis (Wolf-Ringwall et al., 2019; Tagawa et al., 2018; Riondato et al., 2021; Rao et al., 2011; Pinheiro et al., 2014; Poggi et al., 2015; Poggi et al., 2017; and Mizutani et al., 2016), two correlated cell size with prognosis (Rao et al., 2011; and Wolf-Ringwall et al., 2019), one correlated peripheral lymphocyte/monocyte ratio with prognosis (Marconato et al., 2015), one correlated bone marrow infiltration measured by flow cytometry with prognosis (Marconato et al., 2013), one looked at evaluating extranodal infiltration by flow cytometry (Joetzke et al., 2012) two correlated specific cellular markers with lymphoma grade (Poggi et al., 2015; and Riondato et al., 2021) and two correlated peripheral regulatory T-cells measured by flow cytometry with lymphoma severity (Baek et al., 2017; and Pinheiro et al., 2014). The significance of these will be assessed below.

We must also take into account the various forms of lymphoma included in the study populations assessed in this review. Four studies included dogs with any form of B-cell lymphoma in their study populations (Rao et al., 2011; Baek et al., 2017; Poggi et al., 2017; and Pinheiro et al., 2014). Four studies only included dogs with multicentric B-cell lymphoma (Wolf-Ringwall et al., 2019; Tagawa et al., 2018; Marconato et al., 2013; and Marconato et al., 2015). These studies are still very relevant to the PICO however due to the high prevalence of multicentric B-cell lymphoma in veterinary practice. The remaining four studies included patients with both B- and T-cell lymphoma, however all had a study population predominantly containing dogs with B-cell lymphoma (Poggi et al., 2015; Mizutani et al., 2016; Joetzke et al., 2012; and Riondato et al., 2021).  Additionally, four studies (Mizutani et al., 2016; Poggi et al., 2015; Poggi et al., 2017; and Rao et al., 2011) did not perform WHO classification on their cases, meaning that there is an unknown mix of B-cell lymphoma subtypes in these studies. We should consider these factors when interpreting the studies, however they likely still provide us with representative information.

### Cellular Markers

Eight of the studies identified an association between specific cellular markers and prognosis in B-cell lymphoma (Wolf-Ringwall et al., 2019; Tagawa et al., 2018; Riondato et al., 2021; Rao et al., 2011; Pinheiro et al., 2014; Poggi et al., 2015; Poggi et al., 2017; and Mizutani et al., 2016). These markers may be useful in providing prognostic information in dogs with B-cell lymphoma by predicting survival, after treatment with cytotoxic drugs, usually based on their percentage expression in the relevant cellular population.

Mizutani et al. (2016) found that patients with lymphoma with a high percent of CD25+ B-cells in peripheral lymph nodes had a significantly decreased progression free survival and a subjectively decreased overall survival time compared to patients with a low percent of CD25+ B-cells. Although this study included dogs with multiple lymphoid malignancies, it predominantly included dogs with high-grade B-cell lymphoma (17/26). This correlation was also found by Wolf-Ringwall et al (2019) who evaluated CD25 expression in dogs with multicentric B-cell lymphoma. CD25 binds the growth factor Interleukin-2 which stimulates clonal expansion and maturation of activated T or B lymphocytes (Lowenthal et al., 1985) which likely explains this relationship. These results suggest that CD25 could represent a useful prognostic marker for dogs with B-cell lymphoma.

Tagawa et al. (2018) assessed the expression of Programmed Death-1 (PD-1) and Cytotoxic T-Lymphocyte Associated Antigen-4 (CTLA-4) in samples taken from the peripheral blood and palpable lymph nodes in dogs with high-grade multicentric B-cell lymphoma. PD-1 and CTLA-4 are immune checkpoint molecules which are believed to be highly expressed on tumour infiltrating and peripheral lymphocytes and help evasion of the host immune system. Dogs with high-grade B-cell lymphoma were found to have up-regulated expression of PD-1 on peripheral and tumour infiltrating lymphocytes and up-regulated expression of CTLA-4 on peripheral CD4+ T-cells. Dogs with CTLA-4 expression below the cutoff value established by the study had significantly longer survival times compared to dogs with CTLA-4 expression above the cutoff value (with the exception of CD8+ CTLA-4+ cells from lymph nodes which did not meet the minimum AUC required). Although increased in dogs with B-cell lymphoma, the expression of PD-1 was not found to reliably correlate with survival times. Although there were certain limitations with this study, such as a small sample size and varying chemotherapeutic protocols, the expression of CTLA-4 on T-cells from peripheral blood and lymph node samples seems to be associated with a poorer prognosis in dogs with multicentric B-cell lymphoma, and further studies with larger populations could help to further refine the cutoff for its use as a prognostic indicator.

Class II MHC is another cellular marker that is primarily expressed on ‘professional’ antigen presenting cells and has been assessed by three of the studies in this review (Pinheiro et al., 2014; Rao et al., 2011; and Wolf-Ringwall et al., 2019). Rao et al. (2011) assessed for a correlation between class II MHC expression and survival in dogs with B-cell lymphoma. They found that dogs with low class II MHC expression had a lower median survival time than dogs with high class II MHC expression. In their study, dogs with low class II MHC expression were 2.9 times more likely to die in any time period compared to high expression. However, Wolf-Ringwall et al. (2019) did not find a correlation between class II MHC expression and outcome in dogs with diffuse large B-cell lymphoma. One possible explanation for this put forward by Wolf-Ringwall et al. (2019) is that class II MHC levels may not be important for survival in some histopathologic subtypes as Rao et al. (2011) did not sub-classify the B-cell lymphomas in their study by histologic subtype. However, diffuse large B-cell lymphoma is the most common type of B-cell lymphoma in dogs, therefore it would be expected that diffuse large B-cell lymphoma would make up a large proportion of the population in the study by Rao et al. (2011).  Pinheiro et al. (2014) found that decreased class II MHC expression was associated with increased time to remission in their study. This supports the findings by Rao et al. (2011), suggesting that low expression of class II MHC is associated with a poorer prognosis. The expression of class II MHC could be a useful prognostic indicator, however further studies to clarify this would be required before implementing it as a prognostic tool.

Ki67 is a marker of cellular proliferation and is frequently used to assess the growth fraction of neoplastic populations (Schlüter et al., 1993). Poggi et al. (2015) and Riondato et al. (2021) both investigated Ki67 as a cellular marker. Poggi et al. (2015) initially assessed the percentage of Ki67+ cells in lymph nodes samples (Ki67%) in dogs with lymphoma (including 65 with B-cell lymphoma) and confirmed that Ki67% is increased in samples from dogs with high-grade lymphoma compared to low-grade. However, for the purposes of this review, only three dogs with low-grade B-cell lymphoma were included in the study population compared to 62 with high-grade B-cell lymphoma. The rest of the study population was made up of dogs with high- and low-grade T-cell lymphoma. Thus, when considering the significance of the study for this review, we need to be aware that this finding should be confirmed in a study with a greater population of dogs with low-grade B-cell lymphoma. Riondato et al. (2021) lead a similar study with an increased sample size and found similar results. In the same study, Poggi et al. (2015) also established a suggested cutoff value for Ki67% of 12.2% to detect high-grade lymphoma which could be useful when classifying lymphoma and the prognostic implication that carries. For dogs with B-cell lymphoma, this should be confirmed before implementation by means of further investigation due to the lack of dogs with low-grade B-cell lymphoma included in the study as mentioned above.

Poggi et al. (2017) then looked to correlate Ki67% with survival in dogs with high-grade B-cell lymphoma. Ki67% was found to significantly influence lymphoma specific survival and dogs with an intermediate Ki67% compared to a high or low Ki67% had significantly longer relapse free interval and lymphoma specific survival. Ki67% was also a significant predictor for 1 and 2 year survival. The authors suggest that this relationship is due to the fact that lymphomas with low proliferation rates (and therefore lower expression of Ki67) exhibit resistance to cycle specific cytotoxic chemotherapy and lymphomas with high proliferation rates (and therefore higher expression of Ki67) are more likely to regrow or acquire further mutations resulting in treatment failure. Poggi et al. (2017) thus suggested that Ki67 could be a useful prognostic indicator for addition to the routine panel of labelling for high-grade B-cell lymphoma with intermediate values of Ki67% being associated with the best prognosis. 

### Cellular Size

It has also been suggested that size of neoplastic cells in samples taken from dogs with B-cell lymphoma could represent a possible prognostic indicator. Rao et al. (2011) classified cells taken from samples from peripheral lymph nodes of dogs with B-cell lymphoma on the basis of size evaluated by flow cytometry. In this study, the median forward scatter of CD21+ cells in the sample was assessed. Large cells were categorised as having a median forward scatter of >720U. Patients in the large cell group were found to be 2.8 times more likely to die in any time period compared to those in the small cell group. It is suggested that these large B lymphocytes correlate to more immature and aggressive neoplastic cells which are negatively associated with survival. Wolf-Ringwall et al. (2019) also characterised CD21+ cells taken from lymph nodes of dogs with multicentric B-cell lymphoma with flow cytometry. A cutoff value for median forward scatter of 720U was also used in this study, with median forward scatter of >720U being used to assign a sample to the large cell group. Wolf-Ringwall et al. (2019) did not find any correlation of cell size with survival, contrasting the findings of Rao et al. (2011). However, the large cell group only comprised of 3.1% (2/64) patients in the study by Wolf-Ringwall et al. (2019), which may explain the lack of statistical correlation. In the study by Rao et al. (2011), still only 6.9% (11/160) of the study population was assigned to the large cell group. As far fewer patients were assigned to the large cell group compared to patients in the small cell group in both studies, cell size measured by this basis may not provide a widely applicable prognostic test for dogs with B-cell lymphoma.  Cell size can also be influenced by the time delay between sampling and flow cytometry, during which cells may swell and not be representative of their true size. Additionally, the methods used for classifying cell size are not transferable between studies, making it difficult to compare between them. The significance of cell size on prognosis is still not clear, however further investigations are warranted for the use of flow cytometry for this purpose.

Bone Marrow Infiltration

A novel use for flow cytometry was employed by Marconato et al. (2013) who looked to correlate bone marrow infiltration by multicentric B-cell lymphoma with time to progression and lymphoma specific survival with a view to use it as a prognostic tool. Bone marrow infiltration was reported as percentage of large CD21+ cells out of total CD45+ cells in the sample. They found that bone marrow infiltration significantly influenced time to progression and lymphoma specific survival, thus suggesting that level of bone marrow infiltration can be used as a prognostic tool in multicentric B-cell lymphoma. They suggested a cutoff of 3.0% bone marrow infiltration to identify dogs with an unfavourable prognosis. Dogs with ≥3.0% bone marrow infiltration had a 3.3 times higher probability of progression and a 3.6 times higher probability of death when compared to dogs with < 3.0% bone marrow infiltration. Analysis of bone marrow infiltration is commonly involved in the staging of canine lymphoma (Marconato, 2011) and this study shows that flow cytometry can be used to assess bone marrow infiltration and give prognostic information relating to this. A cut-off value is also established which could be further refined and validated following further investigation and use.

Extranodal Infiltration

Joetzke et al. (2012) used the ratio between T and B lymphocytes (log (T:B)) established by flow cytometry to differentiate between lymphoma infiltrated and healthy samples from blood, bone marrow, liver and spleen.  Using this method they found that mean log (T:B) values in all samples were significantly lower in dogs with B-cell lymphoma compared to healthy control dogs. The authors suggest that the log (T:B) could be used as an indicator of the degree of lymphoma cell infiltration to predict prognosis, however this would need to be investigated in further studies as it has not been confirmed that log (T:B) correlates with prognosis, nor is it clear that that the B and T-cells are the neoplastic cells in question. However, the ability of log (T:B) to predict lymphoma infiltration would suggest that it may correlate with prognosis. Although log (T:B) measured by flow cytometry has not yet been established as a prognostic measure in canine lymphoma, this study has shown that it may prove useful in the future and further studies should focus on establishing its use.

### Peripheral Lymphocyte/Monocyte Ratio

Marconato et al. (2015) suggested that peripheral lymphocyte/monocyte ratio (LMR) determined by flow cytometry may provide useful prognostic information in dogs with diffuse large B-cell lymphoma. This has previously been shown in humans (Li et al., 2014). They found that the probability of lymphoma progression was 3.691 times higher in dogs with an LMR ≤1.2 compared with dogs with an LMR >1.2. As well as this, the probability of death due to lymphoma was 4.131 times higher in dogs with an LMR ≤1.2 compared with dogs with an LMR >1.2. This technique could be useful as it makes use of a peripheral blood sample rather than aspirates of lymph nodes which may be less stressful for the dog. However, this information could be obtained using a standard analyser rather than requiring flow cytometry - a more complex technique. While LMR looks to be a promising addition to the range of prognostic indicators assessed by flow cytometry, it is not clear if there are advantages to calculating this using flow cytometry rather than a standard analyser.  While this study used a chemoimmunotherapy protocol to treat the patients, further investigations could assess the prognostic value of LMR in patients undergoing traditional chemotherapy.

Peripheral Regulatory T-cell (Treg) Population

It has been suggested that Tregs are involved in host immunosuppression as a protective mechanism employed by tumours in order to escape the host protective immune system (Yang et al., 2004). Baek et al. (2017) thus investigated the percentage of Tregs in total lymphocytes of the peripheral blood with flow cytometry in dogs with B-cell lymphoma of different stages. They found that the percentage of Tregs in peripheral blood was increased in dogs with lymphoma compared to healthy control dogs. They also found that dogs with WHO stage V lymphoma had increased percentage of Tregs in peripheral blood compared to dogs with WHO stage IV lymphoma. This suggests that percentage of Tregs in peripheral blood could be used as a possible prognostic indicator due to its correlation with lymphoma grade. However, an association of Treg percentage and survival was not assessed so we cannot make this assumption. As well as this, sample size was small and only dogs with WHO stage IV and V lymphoma were sampled, not giving a representative sample across different stages.

Pinheiro et al. (2014) assessed the expression of FOXP3 and Helios in samples from dogs with B-cell lymphoma.  In these dogs, FOXP3 and Helios expression is used as a surrogate for levels of regulatory T-cells in the affected lymph nodes. They found that increased FOXP3 expression in patients was associated with poorer progression free survival and overall survival times. Patients with higher Helios expression also had a shorter progression free survival time. This study provides stronger evidence for the use of Treg populations in lymph nodes as a prognostic indicator in dogs with B-cell lymphoma. Peripheral Treg percentage may have a role as a prognostic indicator, however its relationship with lymphoma grade and survival should be further characterised.

### Conclusion

There are several promising flow cytometric prognostic indicators and as our range of antibodies to cellular markers grows in dogs, it is likely that further targets will be revealed. From the studies evaluated, there is currently good evidence for the use of percentage expression of CD25 and Ki67 cellular markers in providing prognostic information in canine B-cell lymphoma. This is due to the presence of more than one good quality study suggesting each of their prognostic values. However, this needs to be translated into clinical practice and their use should be evaluated on a wider scale, including the provision of cutoff values for estimates of survival or response to treatment.

There is also evidence for the prognostic value of flow cytometric analysis of bone marrow infiltration, expression of class II MHC and CTLA-4, peripheral lymphocyte / monocyte ratio, nodal regulatory T-cell populations and the ratio between T and B lymphocytes in extranodal locations, however these need to be further investigated before introduction to clinical practice in order to confirm their value. This should involve more studies assessing these prognostic indicators with larger sample sizes. Furthermore, the use of cellular size and peripheral regulatory T-cell populations as prognostic indicators cannot be confirmed from the studies evaluated due to the lack of good quality evidence on the subject including limitations with sampling. While they may still hold value in providing prognostic information, this needs to be assessed by further studies on this topic.

Flow cytometry offers the evaluation of numerous variables which can be used to help assess the prognosis of canine patients with B-cell lymphoma and its use will only grow as more canine antibodies against cellular targets become available.

## Methodology Section

**Search Strategy:** 

Databases searched and dates covered:	CAB Abstracts on OVID Platform 1973–Week 25 2021 PubMed 1973–2021 Web of Science 1973–2021
Search strategy:	CAB Abstracts: (dog or dogs or canine* or canis) or exp dogs/ or exp canis/ (((B-cell or B) and (lymphoma* or lymphosarcoma* or lymphoprolifer*)) or BCL) ((flow and cytomet*) or FC) 1 and 2 and 3 PubMed: ((dog or dogs or canine* or canis) AND (((B-cell or B) AND (lymphoma* or lymphosarcoma* or lymphoprolifer*)) or BCL) AND ((flow AND cytomet*) or FC)) Web of Science: ((dog or dogs or canine* or canis) AND (((B-cell or B) AND (lymphoma* or lymphosarcoma* or lymphoprolifer*)) or BCL) AND ((flow AND cytomet*) or FC))
Dates searches performed:	27 Oct 2021

**Exclusion / Inclusion Criteria:** 

Exclusion:	Wrong species Not B-cell lymphoma No full text available Not relevant to PICO Non-English language publications Published more than 25 years ago Single case reports
Inclusion:	Correct species B-cell lymphoma English language papers relevant to PICO Full text available Papers relevant to veterinary medicine

**Search Outcome:** 

Database	Number of results	Excluded – Not relevant to PICO	Excluded – Wrong species	Excluded – Not B-cell lymphoma	Excluded – Single case report	Total relevant papers
CAB Abstracts	91	78	0	1	3	9
PubMed	137	123	0	5	1	8
Web of Science	132	116	2	5	2	7
Total relevant papers when duplicates removed						12

## Conflict of Interest

The authors declare no conflicts of interest.

## Disclaimer

Knowledge Summaries are a peer-reviewed article type which aims to answer a clinical question based on the best available current evidence. It does not override the responsibility of the practitioner. Informed decisions should be made by considering such factors as individual clinical expertise and judgement along with patient’s circumstances and owners’ values. Knowledge Summaries are a resource to help inform and any opinions expressed within the Knowledge Summaries are the author’s own and do not necessarily reflect the view of the RCVS Knowledge. Authors are responsible for the accuracy of the content. While the Editor and Publisher believe that all content herein are in accord with current recommendations and practice at the time of publication, they accept no legal responsibility for any errors or omissions, and make no warranty, express or implied, with respect to material contained within. For further information please refer to our Terms of Use.

## References

[BIBR-1] Baek D.S., Chung T.H., Kim Y.H., Oh S.K., So K.M., Park C. (2017). Changes in regulatory T-cells in dogs with B-cell lymphoma and association with clinical tumour stage. Veterinarni Medicina.

[BIBR-2] Fournel-Fleury C., Magnol J.P., Bricaire P., Marchal T., Chabanne L., Delverdier A., Bryon P.A., Felman P. (1997). Cytohistological and immunological classification of canine malignant lymphomas: Comparison with human non-Hodgkin's lymphomas. Journal of Comparative Pathology.

[BIBR-3] Joetzke A.E., Eberle N., Nolte I., Mischke R., Simon D. (2012). Flow cytometric evaluation of peripheral blood and bone marrow and fine-needle aspirate samples from multiple sites in dogs with multicentric lymphoma. American Journal of Veterinary Research.

[BIBR-4] Li Y.-L., Pan Y.-Y., Jiao Y., Ning J., Fan Y.-G., Zhai Z.-M. (2014). Peripheral blood lymphocyte/monocyte ratio predicts outcome for patients with diffuse large B-cell lymphoma after standard first-line regimens. Annals of Hematology.

[BIBR-5] Lowenthal J.W., Zubler R.H., Nabholz M., MacDonald H.R. (1985). Similarities between interleukin-2 receptor number and affinity on activated B and T lymphocytes. Nature.

[BIBR-6] Marconato L. (2011). The staging and treatment of multicentric high-grade lymphoma in dogs: A review of recent developments and future prospects. The Veterinary Journal.

[BIBR-7] Marconato L., Martini V., Aresu L., Sampaolo M., Valentini F., Rinaldi V., Comazzi S. (2013). Assessment of bone marrow infiltration diagnosed by flow cytometry in canine large B-cell lymphoma: Prognostic significance and proposal of a cut-off value. The Veterinary Journal.

[BIBR-8] Marconato L., Martini V., Stefanello D., Moretti P., Ferrari R., Comazzi S., Laganga P., Riondato F., Aresu L. (2015). Peripheral blood lymphocyte/monocyte ratio as a useful prognostic factor in dogs with diffuse large B-cell lymphoma receiving chemoimmunotherapy. The Veterinary Journal.

[BIBR-9] Mizutani N., Goto-Koshino Y., Tsuboi M., Kagawa Y., Ohno K., Uchida K., Tsujimoto H. (2016). Evaluation of CD25-positive cells in relation to the subtypes and prognoses in various lymphoid tumours in dogs. Immunopathology.

[BIBR-10] Owen L.N., Organization World Health, Owen N. (1980). TNM Classification of Tumours in Domestic Animals. https://apps.who.int/iris/bitstream/handle/10665/68618/VPH_CMO_80.20_eng.pdf.

[BIBR-11] Pinheiro D., Chang Y.-M., Bryant H., Szladovits B., Dalessandri T., Davison L.J., Yallop E., Mills E., Leo C., Lara A., Stell A.Polton, Garden O.A. (2014). Dissecting the regulatory microenvironment of a large animal model of non-Hodgkin lymphoma: Evidence of a negative prognostic impact of FOXP3+ T-cells in canine B-cell lymphoma. PloS One.

[BIBR-12] Poggi A., Miniscalco B., Morello E., Comazzi S., Gelain M.E., Aresu L., Riondato F. (2015). Flow cytometric evaluation of ki67 for the determination of malignancy grade in canine lymphoma. Veterinary and Comparative Oncology.

[BIBR-13] Poggi A., Miniscalco B., Morello E., Gattino F., Delaude A., Ferrero Poschetto L., Aresu L., Gelain M.E., Martini V., Comazzi S., Riondato F. (2017). Prognostic significance of Ki67 evaluated by flow cytometry in dogs with high‐grade B‐cell lymphoma. Veterinary and Comparative Oncology.

[BIBR-14] Ponce F., Marchal T., Magnol J.P., Turinelli V., Ledieu D., Bonnefont C., Pastor M., Delignette M.L., Fournel-Fleury C. (2010). A morphological study of 608 cases of canine malignant lymphoma in France with a focus on comparative similarities between canine and human lymphoma morphology. Veterinary Pathology.

[BIBR-15] Rao S., Lana S., Eickhoff J., Marcus E., Avery P.R., Morley P.S., Avery A.C. (2011). Class II major histocompatibility complex expression and cell size independently predict survival in canine B‐cell lymphoma. Journal of Veterinary Internal Medicine.

[BIBR-16] Riondato F., Martini V., Melega M., Poggi A., Miniscalco B. (2021). Variation of apoptotic and proliferative activity among lymphoma subtypes in dogs: A flow cytometric study. Research in Veterinary Science.

[BIBR-17] Schlüter C., Duchrow M., Wohlenberg C., Becker M.H., Key G., Flad H.D., Gerdes J. (1993). The cell proliferation-associated antigen of antibody Ki-67: a very large, ubiquitous nuclear protein with numerous repeated elements, representing a new kind of cell cycle-maintaining proteins. Journal of Cell Biology.

[BIBR-18] Tagawa M., Kurashima C., Takagi S., Maekawa N., Konnai S., Shimbo G., Matsumoto K., Inokuma H., Kawamoto K., Miyahara K. (2018). Evaluation of costimulatory molecules in dogs with B-cell high grade lymphoma. PloS One.

[BIBR-19] Vail D.M., Michels G.M., Khanna C., Selting K.A., London C.A. (2010). & Veterinary Cooperative Oncology Group. Veterinary and Comparative Oncology.

[BIBR-20] (2019). Prospective evaluation of flow cytometric characteristics, histopathologic diagnosis and clinical outcome in dogs with naïve B‐cell lymphoma treated with a 19‐week CHOP protocol. Veterinary and Comparative Oncology.

[BIBR-21] (2004). Intratumoral administration of dendritic cells overexpressing CCL21 generates systemic antitumor responses and confers tumor immunity. Clinical Cancer Research.

